# Evidence of Environmental Changes Caused by Chinese Island-Building

**DOI:** 10.1038/s41598-019-41659-3

**Published:** 2019-03-28

**Authors:** Leland Smith, Peter Cornillon, Don Rudnickas, Colleen B. Mouw

**Affiliations:** 1 Georgetown University Law Center, Washington, DC USA; 2 Graduate School of Oceanography, University of Rhode Island, Narragansett, RI USA; 3 U.S. Coast Guard International Ice Patrol, New London, CT USA

## Abstract

This paper quantifies environmental effects of island-building operations in the South China Sea, which result from dredging and can negatively impact marine flora, fauna, and ecosystems. The extent of the damage caused by island-creation is believed to be large, as the South China Sea reefs support the largest concentration of marine biodiversity on Earth. Through use of satellite imagery, we investigate the island-construction on Mischief Reef in the South China Sea, showing backscatter increases of up to 350% in waters surrounding the reef, with plumes of excess sediment exceeding 250 km^2^ at times during island-construction, and the cumulative area impacted by dredging exceeding 1,200 km^2^. Comparison of satellite-derived chlorophyll-a, backscatter, absorption and remote sensing reflectance at 412 nm suggest that dredging activities led to a decrease in biological health of the region resulting from the smothering of natural benthic habitats and reef complexes with sediment. We anticipate this ex post facto quantification of the connectivity between island-construction, large particulate plumes and a decrease in absorption related to marine life in the water column to establish a starting point for further study into ecosystem impact. The potential associations between these damages and a long-term reduction in ocean life and resources could serve inter-governmental bodies with a baseline metric for evaluating the level of damage caused. This may result in both forward-looking deterrent policies that limit island-building as well as backward-looking compensation.

## Introduction

**Figure 1:**
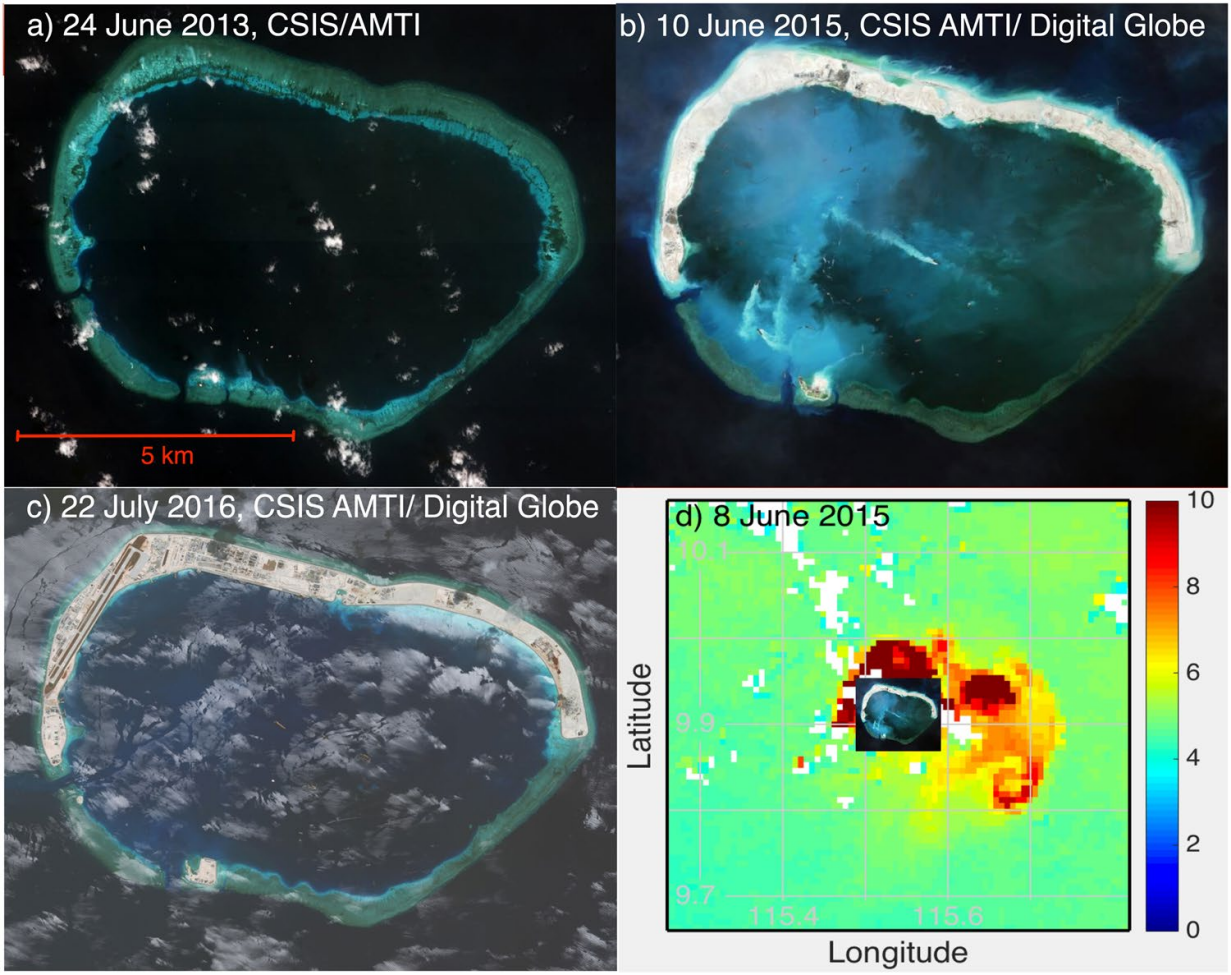
Progression of island building and ocean changes at Mischief Reef. High resolution imagery from the Asia Maritime Transparency Initiative at the Center for Strategic and International Studies^14^ shows (**a**) untouched Mischief Reef in 2013 Image© 2013 DigitalGlobe, Inc, (**b**) significant dredging activity evident in 2015, Image© 2015 DigitalGlobe, Inc (**c**) land development evident by 2016, Image© 2016 DigitalGlobe, Inc and, (**d**) Our analysis of Ocean Color imagery from the MODerate-resolution Imaging Spectroradiometer (MODIS)-Aqua indicates a large backscattering plume coincident with a time of high dredging activity evidenced in (**b**). The inset shows (**b**) to the scale of the ocean color image. [NASA Goddard Space Flight Center, Ocean Ecology Laboratory, Ocean Biology Processing Group; (2015): MODIS Aqua Ocean Color Data, NASA OB.DAAC. https://oceandata.sci.gsfc.nasa.gov/cgi/getfile/http_manifest.txt?h=ocdist103&p=/data2/c080a010e2fe8158. Accessed on 2017/04/27].

Artificial island-creation generally involves cutter suction dredging (CSD) and/or trailing hopper dredging (THD) often followed by airfield construction^1,2^. This activity allows nations to project military power by adding armaments on these new islands, and to protect their highly important fishing fleets^3^. In the South China Sea (SCS) between 2013 and 2017, China built 3,200 acres of new land, Vietnam built 120 acres of new land, and Taiwan built eight acres of new land^4^. Given this level of activity in the SCS and the fact that environmental alterations resulting from dredging can include the loss of species in the benthic community^5^, it is natural to ask: what impact do these activities have on the surrounding ocean? The potential causality between Chinese island-building operations and environmental degradation is potentially significant, as the Spratly Islands in the SCS form a distinct marine ecosystem, which serves as a significant source of larvae for regional coral reefs^6,7^. Increasing demand for fish in the SCS has led to political tensions and even violence as millions of people in the region rely on fishing for food and work^8^. Further complicating these pressures, the preservation of the transport connectivity between living organisms, as well as, pollutant dispersion occurs across sub-regions and across national boundaries within the SCS^2^. Nations outside the SCS are also impacted by the ecology therein as the sea sits on the western edge of the Coral Triangle, which hosts the largest concentration of marine biodiversity on Earth including many threatened species^9^. Although numerous studies have examined the impact of dredging generally^10,11^, safety concerns resulting from the tense political climate in the SCS prevent robust *in-situ* measurements near the sites of recent land-building operations in that region. The work presented herein, based on a case study of Mischief Reef (9.90°N, 115.54°E) (Fig. 1a–c) through December 2018, extends the work begun by [^12^, BH2016 hereafter] in which they document the extent of dredge spoil plumes from the commencement of dredging through December 2015. Of particular interest are signs of a possible biological impact resulting from the dredging. Mischief Reef was selected due to the large amount of dredging that has taken place there −1,379 acres of land reclamation, and the construction of a 2,644 m by 55 m runway (Fig. 1c)^13,14^.

The dates on which dredging operations began and ended do not appear to be publicly available. For the purpose of this study, we bounded the starting date based on analysis of Landsat imagery and imagery hosted on the Asia Maritime Transparency Initiative, Mischief Reef Tracker web site [https://amti.csis.org/mischief-reef/ as of 26 October 2017] of The Center for Strategic and International Studies. Mischief Reef was largely submerged in 2014, above the waterline in 2015 following commencement of dredging, and inhabited by living quarters and an airstrip in 2016 (Fig. 1a–c from the Mischief Reef Tracker web). These observations are consistent with the increase in area of impacted water documented below.

## Methods

### Analysis of Dredging

Landsat 7 Enhanced Thematic Mapper Plus (ETM+), Landsat 8 Operational Land Imager (OLI), and Sentinel 2A imagery (level 1 C) [Accessed via the United States Geological Survey (USGS) EarthExplorer web page https://earthexplorer.usgs.gov/] were examined to track the progress of Mischief Reef island building. Utilizing visual interpretation and manual delineation in each usable image, the number of ships present in the lagoon was counted, the number of active dredges was estimated, the exposed land area was quantified, and a dredging rate was determined from January 2014 through December 2017. Imagery with less than 50% cloud cover over the reef was downloaded and compiled into a multi-spectral image using the layer stack functionality in ERDAS Imagine 2016.

Mischief Reef is located in Path 119, Row 53 of the Landsat orbit. Landsat 7 was launched in 1999 and Landsat 8 in 2013. Both Landsat satellites have a repeat cycle of 16 days, passing the study area at approximately 0240Z but Landsat 7 and 8 are offset so that there is a combined temporal resolution of 8 days. Both the ETM+ and OLI sensors are multi-spectral with 30 m spatial resolution. For true color: bands 3, 2, and 1 in red, green and blue (RGB) were utilized for ETM+ and bands 4, 3, and 2 in RGB for OLI. We also utilized the Panchromatic band (band 8) with 15 m resolution available on both sensors. In 2003, Landsat 7’s Scan Line Corrector (SLC) failed resulting in bands of omitted pixels approximately 120 meters (4 pixels) wide, resulting in a source of error for the ship count. Images where omitted pixels were parallel to and overlaying the coastline were not used for the area delineation.

**Figure 2:**
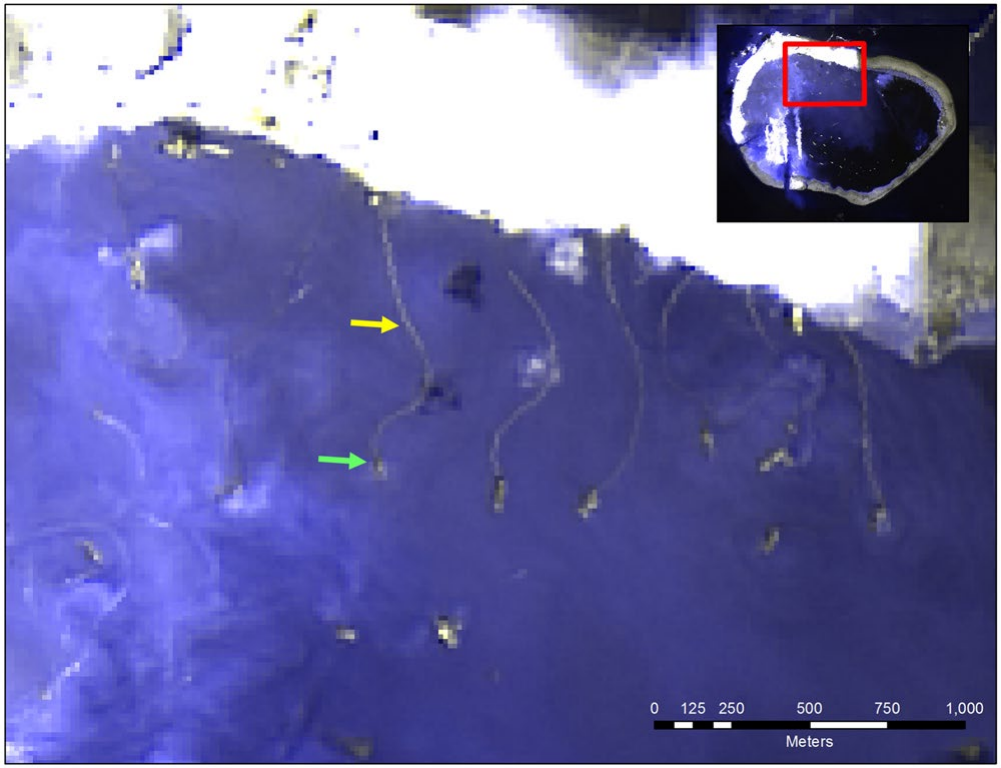
Cutter Suction Dredges identified by dredge pipes by OLI using bands 8-8-2 in RGB on May 6, 2015. The green and yellow arrows point to a sample dredge and pipeline, respectively. Landsat-8 imagery courtesy of the U.S. Geological Survey. This image was generated using ERDAS Imagine 2016®, which is a software product owned by Intergraph Corporation doing business as Hexagon Geopatial. © 2016 Hexagon and/or its subsidiaries and affiliates. All rights reserved.

**Figure 3:**
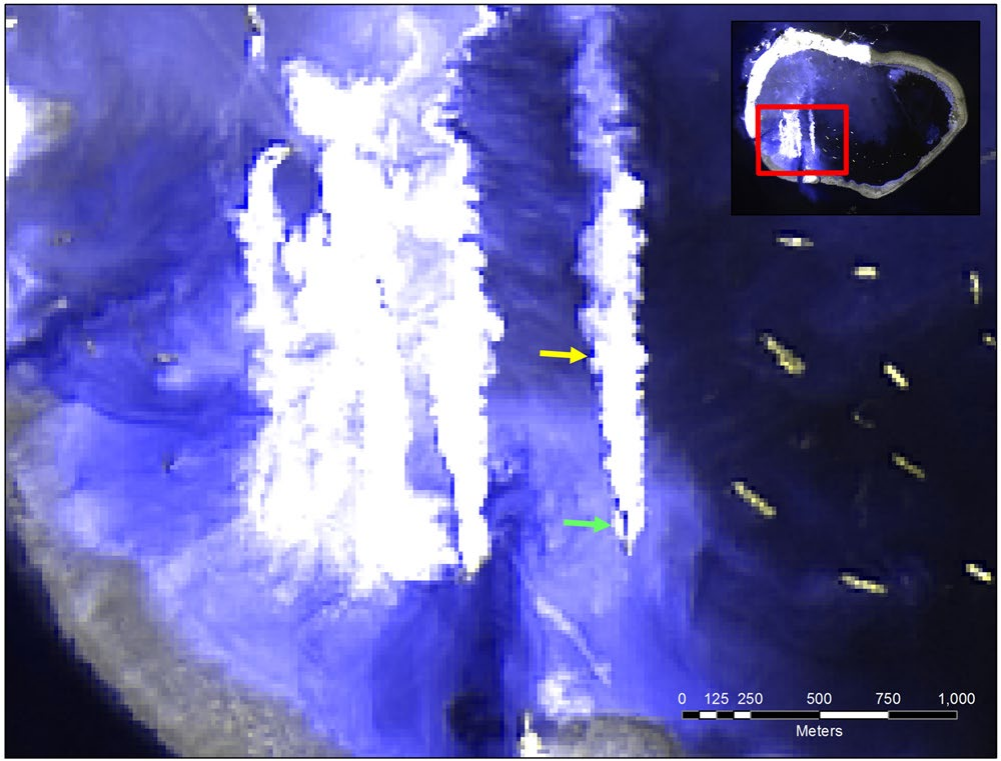
Trailing Hopper Dredges identified by along-side plumes by OLI using bands 8-8-2 in RGB on May 6, 2015. The green and yellow arrows point to a sample dredge and resulting plume, respectively. Landsat-8 imagery courtesy of the U.S. Geological Survey. This image was generated using ERDAS Imagine 2016®, which is a software product owned by Intergraph Corporation doing business as Hexagon Geopatial. © 2016 Hexagon and/or its subsidiaries and affiliates. All rights reserved.

Sentinel 2A was launched in 2015 with a 10 day repeat cycle. Passage over the study area was at approximately 0300Z. Bands 3, 2, and 1 in RGB with 10 m resolution were used for true color imagery. Of the 214 total Landsat and Sentinel frames available, 138 were sufficiently cloud-free to be analyzed. Each image was visually examined in True Color and Panchromatic bands to conduct a count of ships within the lagoon. A second count of ships actively dredging was conducted. Cutter Suction Dredges were identified by the large dredge pipelines leading toward the reef (Fig. 2). Trailing Hopper Dredges were identified as vessels within the lagoon coincident with large plumes alongside (Fig. 3). The spatial resolution of the satellites used prohibited a more detailed identification by gear on deck as well as identification of dredges not engaged in dredging; i.e., the count of dredges involved in island building at Mischief Reef is a lower bound on the actual number.

The spatial resolution of the images was a source of error inherent in this method as the resolution was not fine enough to distinguish vessels that may have been anchored (or “rafted”) alongside each other. A high resolution Digital Globe image from April 2016 was used to ground-truth the types of dredges present and many vessels were observed to be anchored in this manner.

The area of the exposed land was manually delineated using the Draw/AOI toolbox in ERDAS Imagine. Exposed land was clearly distinguishable spectrally from submerged land by strong reflectance, showing bright white in the imagery and strongly contrasting with the surrounding water. By April 30, 2016, no further dredging was identified and the delineation interval was expanded to every six months with no new land area being detected through November 2017. The manual delineation process is subject to error due to the analyst’s interpretation of the imagery. To minimize this, the same analyst conducted every delineation to ensure consistency.

### Measures of Impact

We examine the impact of dredging from two differing perspectives. First, we determine, as a function of time, the area for which backscatter from the upper ocean, in the 412 nm spectral band of the MODerate-resolution Imaging Spectroradiometer (MODIS), exceeds a threshold determined from a region far from Mischief Reef. This provides for an estimate of the impacted area and how this area changes with time. Second, we examine the change in the spectral characteristics of the waters in the vicinity of the reef as a function of distance from the reef. This allows us to separate potential biological impacts from those that are purely physical both in the lagoon and outside of the reef. It is important to stress that these are both water column measurements, they do not provide an estimate of specific corals that have been smothered or otherwise impacted by the dredging. Taken together, however, they do point to a significant biological impact as will be shown below. Also important, only MODIS data were used for the measures of impact hence the results are consistent in time.

#### Area Impacted Determined from Backscatter

We use backscatter, estimated with the Generalized Inherent Optical Properties (GIOP) semi-analytical algorithm^15^ applied to spectral radiances obtained from MODIS-Aqua, to estimate the area impacted. Although the method used differs from that of BH2016, the two approaches agree quite well with regard to the timing of events. This study therefore serves to corroborate the work of BH2016, as well as to provide detail on ship activity and to extend the period covered through December 2018; the study of BH2016 covers the period through December 2015.

To allow for a historical perspective, as well as to provide the data required for this study, every Level 2 (L2) [“Level-2” refers to the processing level of the data, a nomenclature used extensively for satellite-derived datasets, although the precise meaning of the level of processing varies by organization. The definition promulgated by the National Aeronautics and Space Administration (NASA) is used here: http://science.nasa.gov/earth-science/earth-science-data/data-processing-levels-for-eosdis-data-products/. Of importance to this discussion is that L2 products are in satellite coordinates and L3 products are in geographic coordinates] daytime granule from January 2013 through December 2018 touching a portion of Mischief Reef was acquired from NASA’s Ocean Color Web site [https://oceancolor.gsfc.nasa.gov/]. The data extracted from each granule for use in this study consisted of the time of the overpass, and, for each pixel in the granule, a measure of the quality of the data (referred to as l2_flags in the dataset), the location of the pixel (latitude and longitude) and the total absorption and total backscatter based on the GIOP model for each of the spectral bands available on MODIS in the visible portion of the spectrum (412, 443, 469, 488, 531, 547, 555, 645, 667, 678 nm). The study area was covered by the satellite at least once per day but, because the spectral channels of interest are in the visible portion of the spectrum, clear views of the reef and surrounding waters were possible only under cloud-free conditions.

Processing began by regridding the L2 absorption and backscatter fields to a 334 × 334 km plate-carrée grid (L3) centered on Mischief Reef with a 750 m grid spacing in longitude and latitude. [750 m resolution was selected so that the absorption and backscatter fields could be compared with those obtained from Visible-Infrared Imager-Radiometer Suite (VIIRS) carried on the Soumi-National Polar-orbiting Partnership (NPP) spacecraft. However, the retrievals from VIIRS proved to be noisier than those from MODIS, so these data were not used in this analysis.] As part of the regridding process, L2 processing (quality) flags were used to mark, for later exclusion, pixels flagged as high radiance, clouds, low water leaving radiance, absorbing aerosols, chlorophyll out of bounds, suspect atmospheric correction or product failure. Not marked were pixels flagged as containing coccolithophores, turbid water, or moderate glint. A land mask was created by summing the remapped 412 nm backscatter images from 2002–2010 (2679 images in total) and thresholding the resulting image at 4.55 m^−1^; values above 4.55 m^−1^ were set to one, land values, the remaining values, ocean, were set to zero. The resulting land mask corresponds well in shape and size with images of Mischief Reef from the Asia Maritime Transparency Initiative, Mischief Reef Tracker web site. The land mask also agreed well with other exposed reefs in the region. No attempt was made to distinguish between land that was above water at high tide but not at low tide. ‘Land’ pixels were marked for exclusion in the subsequent analysis. For each remapped image, the number of pixels not marked for exclusion, the sum of the backscatter (or absorption) values of these pixels and the sum of the square of the backscatter values was determined for each 1 km annular ring (0–1 km, 1–2 km, 2–3 km, …) centered on Mischief Reef from 1 to 100 km. This allowed for the determination of the mean and variance for any period and/or group of annular rings of interest.

To determine the impacted area, a mean background backscatter value for each visible spectral band in each image was determined for an annulus from 31 to 50 km from the reef, an area not impacted by dredging as will be shown below. The number of pixels in an annulus from 7 to 35 km, which exceeded the mean background backscatter by 30% for the given spectral band, was then multiplied by the area of a pixel yielding the impacted area. Masked pixels may have been impacted but are not counted hence the estimate of the impacted area for a plume is a lower bound on the actual impacted area for that day. Furthermore, the lagoon within Mischief Reef was not included in this calculation because the value within the lagoon often exceeded that of the background in the pre-dredging period as well as, of course, during and following dredging.

In order to estimate the area of the seafloor potentially impacted by sinking sediments, the mean backscatter of pixels, marked as impacted, was determined for 2008 and 2015. The mean backscatter of impacted pixels is given by:

1 $$\overline{CB{S}_{i,j}}=\frac{{\sum }_{k=1}^{N}B{S}_{i,j,k}\,\ast \,Impacte{d}_{i,j,k}}{{\sum }_{k=1}^{N}Clea{r}_{i,j,k}}$$

where *N* is the number of satellite passes for the period of interest, *BS*_*i*, *j*,*k*_ is the backscatter for the (*i*, *j*)^*th*^ pixel of the *k*^*th*^ image,

$$Impacte{d}_{i,j,k}=\{\begin{array}{ll}1\, & {\rm{if}}\,B{S}_{i,j,k} > 1.3\,\ast \,Backgroun{d}_{i,j,k}\\ 0 & \,{\rm{otherwise}}\,\end{array}$$

*Background*_*k*_ is the mean background backscatter as described above for the given image, and

$$Clea{r}_{i,j,k}=\{\begin{array}{ll}1 & {\rm{if}}\,{\rm{there}}\,{\rm{is}}\,{\rm{an}}\,{\rm{acceptable}}\,{\rm{value}}\,{\rm{for}}\,{\rm{pixel}}({\rm{i}},{\rm{j}}){\rm{of}}\,{\rm{the}}\,{{\rm{k}}}^{{\rm{th}}}\,{\rm{image}},\\ 0 & {\rm{otherwise}}\end{array}$$

The number of impacted pixels was determined for each image with at least 100 clear pixels in the background area and at least one clear pixel between 7 and 35 km from the reef center. Monthly means of the various quantities were determined by averaging the quantity over all images for the given month following Eq. 1. Finally, we note that the nearest reefs with surface outcroppings, which are approximately 40 km away, show no connection to the plumes emanating from Mischief Reef (Fig. 1d).

#### Biological Consequences

**Figure 4:**
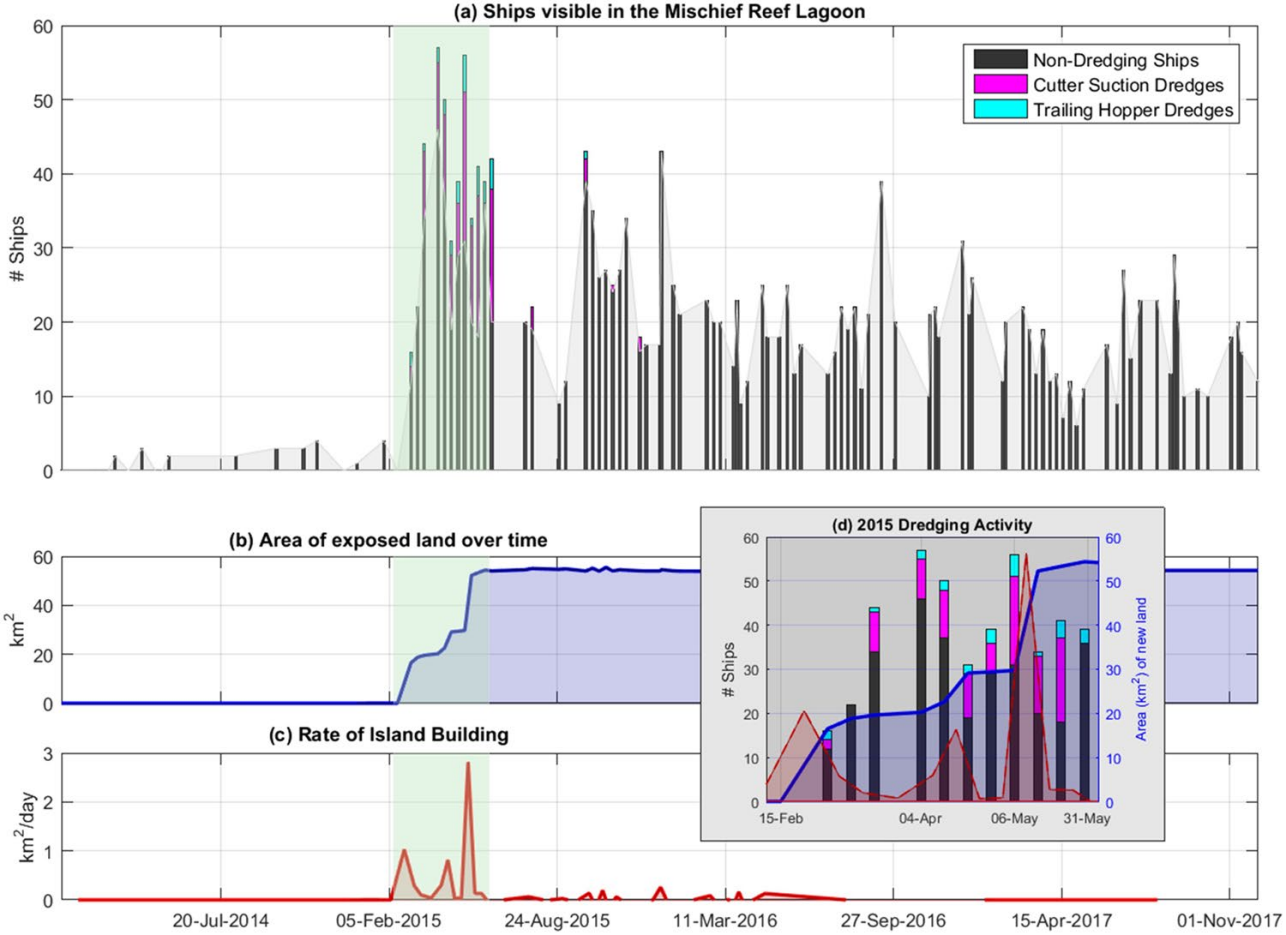
The results of the Landsat and Sentinel image analysis. (**a**) Shows the number of ships (dredging and non-dredging) in the lagoon from January 2014 through December 2017. (**b**) Shows the land area of the island building project. (**c**) Is the rate of dredging (calculated as the change in land area between usable satellite images). (**d**) Is a subset of the high activity dredging timeframe from February through May 2015 identified in (**a**–**c**) by the green shading.

To estimate the potential longer-term biological impact of dredging, we again use MODIS data from Aqua, this time chlorophyll, estimated with the Ocean Color Index (OCI) algorithm^16^, and remote sensing reflectance measured at 412 nm. These data are accessed separately from the GIOP data, although from the same site (the NASA Ocean Color Web site). We again downloaded every L2 daytime OCI granule from July 2002 through December 2018, which touched a portion of Mischief Reef, and followed the same preprocessing steps as described for the GIOP data; i.e., the OCI data were regridded to the same 750 m grid, masked using the same flags (although the values for the OCI product) and then binned into 1 km annular rings centered on Mischief Reef. The same land mask was also used. To serve as points of reference, Fig. 4 displays the number of ships, land area of the island building project, and rate of dredging as a function of time.

#### Choice of Spectral Band

**Figure 5:**
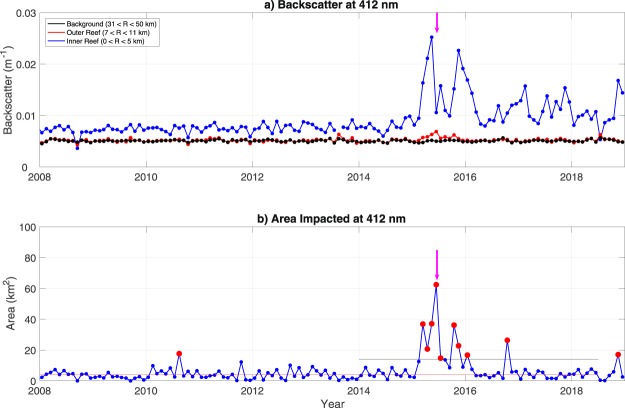
Monthly (**a**) backscatter average in the vicinity of Mischief Reef and (**b**) area impacted in the annulus between 7 and 30 km. Magenta arrows are the values for 8 June 2015. The horizontal red line in the lower panel is the mean area ‘impacted’, resulting from natural variability in the system, prior to 2013 and the gray line is 3 standard deviations above this mean.

**Figure 6:**
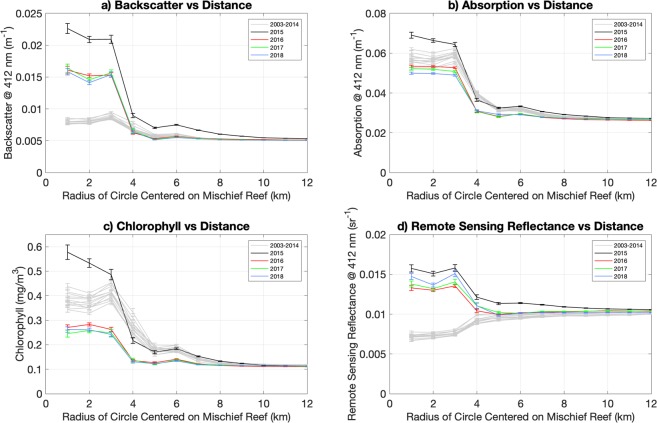
Ocean color parameters by time period as a function of distance from Mischief Reef. (**a**) Backscatter at 412 nm, (**b**) Absorption at 412 nm, (**c**) Chlorophyll Concentration and (**d**) Remote Sensing Reflectance at 412 nm. All are averaged over 1 km annular rings as a function of distance from the center of Mischief Reef for one-year periods starting with 1 January 2003. Lines for years prior to 2015 are shown in gray, the line for 2015 - the period of significant dredging - is shown in black and lines for the period following significant dredging are shown in color.

A clear increase in backscatter during 2015 was seen across all visible spectral bands (412, 443, 469, 488, 531, 547, 555, 645, 667, 678 nm). We present the results for the 412 nm band as this wavelength captures impacts from phytoplankton and sediments (non-algal particles) and the red bands traditionally used to detect turbidity were not reliable (Barnes and Hu, 2016). The monthly mean backscatter, is shown in Fig. 5a for each of three regions: the inner reef (within 5 km of the reef center – 115.54°E, 9.91°), the outer reef (the region immediately outside of the reef – between 7 and 11 km of the reef center), and a background region (between 31 and 50 km of the reef center). Annual means of ccean color parameters as a function of distance from Mischief Reef are shown in Fig. 6 at 412 nm.

## Results

### Dredging Time Line

Dredging and island building on Mischief Reef began in late January 2015 and the reef had reached it’s maximum area (20.94 km^2^) by May 30, 2015 (Fig. 4). Dredging was observed after May but no increase in land area was detected, thus it is assumed that this activity was focused on increasing the height of the land, a dimension not measured by this study. The first exposed land was observed on January 30, 2015 (0.01 km^2^). Ships were observed in the lagoon starting on March 16, 2014 and the first dredging activity (identified by suction hoses and/or sediment plumes alongside vessels in the lagoon) was observed on March 3, 2015. The final satellite image with identified dredging activity was from November 30, 2015. The small (<0.3 km^2^ day^−1^) rates of island building between June 2015 and September 2016 are attributed to slight variations in the delineation process as well as a slight shrinking of the land area as the edges were constructed into a well-defined sea wall.

### Impact Time Line

Figure 5b shows the monthly mean impacted area. The impacted area indicated with the red dot at the time of the short magenta arrow is for the 8 June 2015 satellite pass and corresponds to the image shown in Fig. 1d. The mean backscatter for this satellite pass corresponds to the value of the curve at the location of the magenta arrow in Fig. 5a. (Note that the impacted region extends beyond the limits of the ‘outer reef’ region.) We attribute the mean impacted area of approximately 4 km^2^ prior to dredging (2003–2013) as natural variability in the data – visual inspection of pre-dredging images showed that’impacted’ pixels were generally randomly scattered across the region or were grouped on the periphery of areas flagged for one of the reasons outlined in Section 2. The monthly averages of the impacted area for the period 2008–2017 peak at 62 km^2^ in June of 2015; the area of the plume on 8 June was 262 km^2^ compared with a pre-dredging background value of approximately 7 km^2^. The yearly averaged monthly plume area from 2015 to 2017 is 3.8 km^2^compared with the mean for 2015 of 23 km^2^. These statistics exclude the area within 7 km of the center of the reef. It is important to stress that these numbers are for plume size averaged over a given month, not the cumulative area covered by plumes, which will be discussed below. Furthermore, these averages do not include potentially impacted areas covered by clouds; i.e., again, these numbers represent lower bounds on the impacted area. We could have estimated the total impacted area by normalizing for the fraction cloud cover in the vicinity of the reef but we decided to present the more rigorous lower bound here.

Prior to dredging activity, backscatter was elevated in the center of the reef (to a radial distance of approximately 5 km), compared to the outer reef region, decaying to a constant background level at approximately 8 km (Fig. 6a). The curves for the pre-dredging years are statistically indistinguishable indicating a stable environment. This changes dramatically for backscatter starting in 2015, with a mean backscatter within the reef increasing by a factor of 3 in 2015 over the pre-2015 level (Fig. 6a) and decaying away from the reef to the background level at a radial distance of 10 km. This coincides with the period of most intense dredging. The relatively small increase in the outer reef area compared to that within the reef is not surprising given that the inner reef area is shallow and nearly enclosed (Fig. 1b) compared to the outer reef area, which is in the open ocean, hence dredged sediments in the water column in the inner reef are not likely to disperse as quickly. In the years (2016–2018) following dredging, backscatter within the reef drops to about twice the pre-2015 level, with little change over the three years, and it falls to the pre-2015 levels immediately outside of the reef.

Similar to backscatter, absorption also shows elevated values within the reef compared to the outer reef region. However, there are differences in the response between these parameters associated with the dredging activity. In 2015, when dredging began, absorption is only slightly higher within the lagoon but statistically identical to that of the previous years outside of the lagoon (Fig. 6b). By contrast, absorption is lower within the reef and immediately outside of the reef, to a distance of approximately 8 km, in the post dredging period, 2016 on, than in the pre-dredging period. The difference in the response of absorption and backscatter during the period of intensive dredging is not surprising. Dredging activities move sediment (non-algal particles), which in this region is sand and calcareous silt. Sand has a large impact on the magnitude of backscatter but a small impact on absorption.

The increase of chlorophyll-a levels (Fig. 6c) in 2015, above pre-dredging levels, suggest that dredging may have stimulated phyotplankton growth. While this is possible, we tend to approach these estimates with caution because of the large concentration of sediments in the water column during dredging, concentrations which may have affected the accuracy of the chlorophyll retrievals. More intriguing is the decline in chlorophyll levels following the cessation of dredging – as dredging activities ceased, and sediments in the water column settled out, the natural habitat had been significantly disrupted, smothered by displaced sediment, evidenced by chlorophyll concentration and absorption falling below the pre-dredging levels. Of particular interest is that the decrease in both of these quantities extends to at least 8 km from the center of the reef, at least 2 km beyond the outer edge of the reef.

Inspecting remote sensing reflectance at 412 nm, also suggests these patterns (Fig. 6d). Prior to dredging, remote sensing reflectance was low in the center of the reef, and higher outside the reef. Remote sensing reflectance is approximately equal to the ratio of backscatter over the sum of absorption and backscatter. Thus, the dramatic increase in backscatter and decreased absorption observed in response to dredging activities significantly increases remote sensing reflectance within the reef.

**Figure 7:**
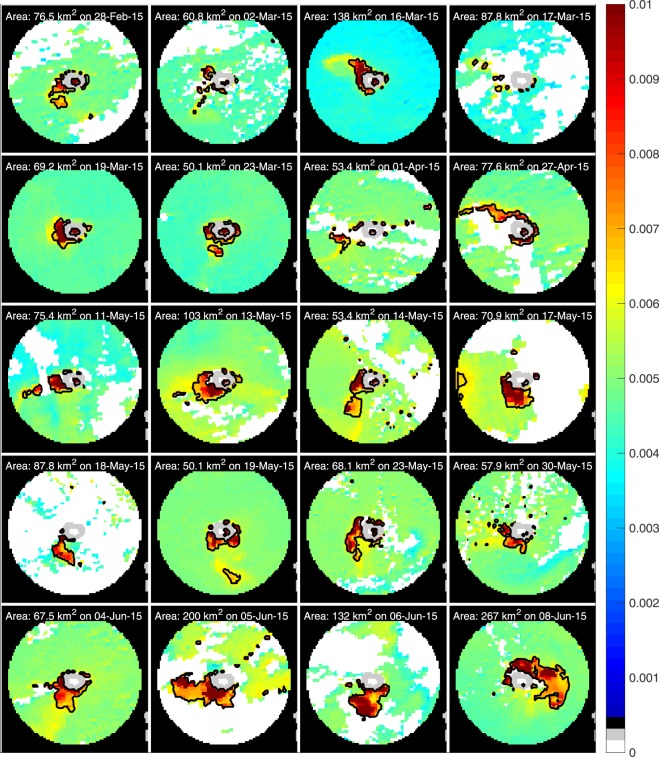
Backscatter at 412 nm within 30 km of Mischief Reef (light gray) on selected days in early 2015. Flagged pixels are indicated in white. The area for which the backscatter >130% of the mean value between 31 and 50 km from the reef center indicated in the heading of each frame. The 0.0064 m^−1^ contours indicated in black. These contours designate the approximate extent of the plume on which the area estimate is based; approximate because the precise contour is determined from the mean concentration of the ‘background’, which changes slightly from image-to-image.

Figure 7 shows the backscatter for the 20 clearest images of the region within 30 km of Mischief Reef between 28 February and 8 June 2015, the last image corresponding to that of Fig. 1d. Waters more than 30 km from the center of Mischief Reef have been masked (black) to focus attention on the plumes arising from dredging. In only one case, 27 April 2015, does the plume of backscatter exceeding the background value (0.0049 m^−1^ for this satellite pass) by a factor of 1.3 (0.0064 m^−1^) extend more than 30 km from the center of Mischief Reef. Of particular interest with regard to these plots is the shape and location of the plume emanating from the reef and, in particular, the rapidity with which it changes. Consider, for example, the four images in the bottom row of the figure,  4–8 June 2015. The plume evolves rapidly from a relatively small plume (68 km^2^) extending to the southwest, to a significantly larger plume (262 km^2^) emanating from the north and wrapping around to the east. Although it is possible that the plume on the 4^*th*^ was advected around the reef contributing to the plume of the 8^*th*^, this is unlikely because the magnitude of the backscatter of the plumes shown (incompletely because of flagged pixels) to the south of the reef for the 5^*th*^ and 6^*th*^ exceeds that of the plume on the 4^*th*^. More likely, the sediment in the plumes sinks relatively quickly, <48 hours, with a concomitant decrease in backscatter and new sediment from the dredging operation is advected away from the reef to form new plumes, which in turn sink relatively quickly. This interpretation is borne out by other pairs of images close in time also displayed in Fig. 7.

**Figure 8:**
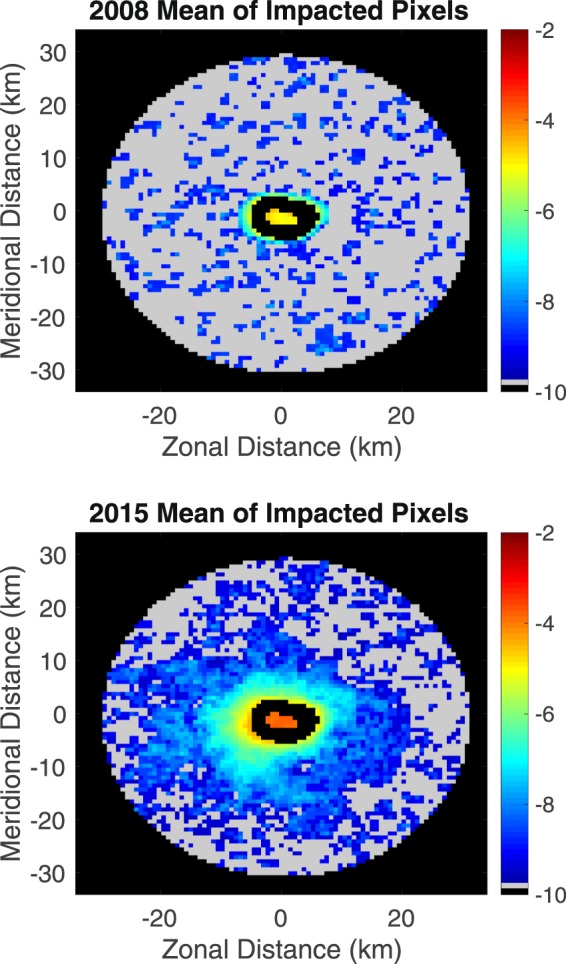
The natural logarithm of the annual mean backscatter of impacted pixels for 2008 (upper panel) and 2015 (lower panel) as a function of distance from Mischief Reef (black elliptical area at the center of the image). Pixels not flagged as impacted at any time during the given year shown in gray.

Figure 8 displays the mean backscatter, determined with Eq. 1, for 2008 (pre-dredging) and the mean backscatter for 2015 (during dredging), both for the same region shown in Fig. 7. Because the dynamic range of the mean backscatter of impacted pixels is large and dominated by the backscatter within the reef, the natural logarithm of the mean values is displayed in Fig. 8. The 2008 mean shows randomly distributed clusters of ‘impacted’ pixels away from the reef. These are likely due to random fluctuations determined from the retrieval algorithm. There is however a region of contiguous ‘impacted’ pixels extending from the reef center to a distance of approximately 2 km beyond the edge of the reef. These are presumably due to sediment eroded into the water column due to natural processes, waves, currents and wind. The mean backscatter of the small clusters of ‘impacted’ pixels away from the reef is less than 1/1000 of the mean backscatter within the reef. By contrast, the 2015 image shows a contiguous area of impacted pixels extending substantially farther into the open ocean, to a distance of approximately 20 km from the center of the reef. The values within the reef are approximately 15 times larger than those for 2008; i.e., those due to natural processes. (The other pre-2015 years are similar to 2008). The mean backscatter beyond the reef decreases by about one order of magnitude per kilometer for the pre-dredging period. The decrease in mean backscatter from the reef edge in 2015 is much slower, approximately an order of magnitude every three kilometers. Beyond about 20 km from the reef center, the distribution of ‘impacted’ patches resembles that of 2008; i.e., the impacted region is limited to within approximately 20 km of the reef center, assuming that the sediment is not advected outside of this region as it sinks to the bottom. This suggests an overall area impacted by dredging operations exceeding 1,200 km^2^, with the impact decreasing rapidly away from the reef. It is difficult to associate a sediment load with the observed backscatter because the characteristics of the sediment in this area are not well known and *in situ* sampling is not possible due to the political situation. These observations must therefore remain qualitative for the present.

## Discussion

Consistent with the observations of BH2016, we see a significant increase in backscatter from the ocean in and around Mischief Reef associated with an increase of sediment in the water column due to dredging. The increase, approximately a factor of three above pre-2015 levels, begins in 2015, consistent with the dredging activity observed in the Landsat and Sentinel data, and, following cessation of dredging falls to twice the pre-2015 level in 2016, remaining constant at that level through 2018. Of particular interest, the heavy load of sediment introduced into the water column appears to have impacted the ecosystem in the region as seen by the decrease in absorption and chlorophyll concentration beginning in 2016 and continuing through 2018, the period for which data were available for this study. Possible causes of this are significant shading of the water column due to the excess suspended sediment from dredging activities. Further, the excess sediment has likely smothered reef complexes from sinking sediment and reduced light to reef complexes and benthic communities. Although the satellite-borne sensor ‘sees’ only the evidence of sediment in the upper ten meters or so of the water column; impact on the seafloor is inferred based on the rapid decrease in the surface concentration; i.e, the assumption is that the sediment is sinking. This is consistent with rapid rate at which the plumes dissipate (Fig. 7) – significantly faster than can be explained by their horizontal diffusion. The image showing the extent of impacted surface water is therefore thought to be a reasonable representation of the seafloor impacted by sinking sediment. As the concentration of the surface distribution of sediments falls off rapidly away from the island, the thickness of the sediment of dredge materials on the seafloor is also thought to fall off rapidly away from the island as would the impact. Figure 7 suggests that the impacted area extends to a distance of approximately 20 km from the center of the reef, an area of approximately 1,200 km^2^, although the impact is likely small on the outer edges of the region. As an aside, we note that, although the area of a given plume is generally a lower bound on the plume size because of potentially impacted pixels being obscured by clouds (or flagged for other reasons mentioned in section 2), the cumulative region, shown in Fig. 8, is likely a reasonable estimate of the impacted region – the only impact clouds would have is on the mean concentration of impacted pixels; i.e., on the volume of sediment sinking to the seafloor at a given location.

As noted above, days in 2015 on which we found significant plumes in the vicinity of Mischief Reef (not shown here) correspond well with those found in the BH2016 study (their Fig. 9). However, the areas of plumes found by BH2016 were approximately 50% larger than the impacted areas we found. This is because of the different measures used. Had the same measure been used here as that used by BH2016, one would expect the impacted areas we find in 2016 and 2017 to be approximately 50% larger than what we show in Fig. 5b. This paper builds upon recent research through concentrated analysis. A recent paper documents the locations of dredging activity across the South China Sea, showcasing the increasing vulnerability of ecologically important areas^17^. Another paper tested the ability of Planet Dove sensors to identify discrepancies in bottom types near many of these dredging sites^18^. Yet another discusses the impacts and widespread use of giant clam chopper boats, which can cause silt-sized material to block light in the water column, at Thitu Island^19^. The amount of silt generated by these operations would have been small relative to the much greater volumes resulting from the dredging and filling operations.

The geo-political significance of the impacts identified above could be large. The SCS is bordered by Taiwan and China to the north, Vietnam to the west, Malaysia and Indonesia to the south, and Brunei and the Philippines to the east. Each of these nations claim full entitlement to resources within the portions of the SCS adjacent to their respective coastlines based on the 1982 United Nations Convention on the Law of the Sea. This law, signed by 167 nations including China and the Philippines, set forth boundary limitations, anti-pollution laws, and many other standards. Pollution by one country in another country’s waters aggravates territorial disputes. The most contested sub-regions are the Spratly and Paracel Islands, which are home to many islands that were uninhabitable to humans. According to expert testimonies during the Philippines v. China Arbitration hearings, Chinese island-building represents the quickest deterioration of coral reefs in human history^20^, a deterioration consistent with the decrease in chlorophyll-a seen from 2016 through 2018 in the lagoon and surrounding waters. Such destruction of coral reefs does not only impact the immediate area, but also reduces commercially vital fish stocks, and the ability of the Pacific Ocean to detoxify waste that can impact climate^21^. Additionally, man-made structures such as airfields and the islands themselves remove reef habitats, may cause leeching, and could cause extinction of local fish, invertebrates, and other critical components of the local ecosystem^22,23^. Primary production is reduced, the reef topography is changed, and the ability to sustain life, as suggested by our results, is likely diminished. In many cases, the island building and harbor digging could be irreversible^24^. Despite multiple studies, court cases, and media reports on the subject of Chinese island-building, little quantification of the extent of the disruption to the surrounding ocean existed prior to this paper. It is therefore difficult for law-makers or multi-lateral regulating organizations to create rules around the topic, or to truly understand the ecological damage that such activity causes. This paper serves as a step toward better understanding by initiating a discussion and providing quantitative measurements from which other scientists can base their analyses in the unfolding story of island-building in the SCS. While this study focuses on one particular reef, there is a much larger practice of this activity currently ongoing.

## Conclusions

Sediment plumes associated with cutter suction and trailing hopper dredging in the vicinity of Mischief Reef are readily detected from satellite-borne sensors measuring in the visible portion of the electromagnetic spectrum as are the ships responsible for dredging. A pixel is identified as impacted by dredging operations if backscatter in the 412 nm spectral band of the sensor exceeds the mean backscatter for the same day in a region between 30 and 50 km from the reef. The contiguous region of such pixels averaged over the period of dredging extends to a distance of approximately 20 km from the reef center, an area in excess of 1,200 km^2^, although the depth of deposited sediment on the seafloor likely decreases rapidly away from the island. This is certainly not the case within the reef where backscatter increases by a factor of approximately three in 2015 and then, in 2016, falls to a factor of approximately twice that at the same locations prior to dredging operations, where it remains through 2018; the inner reef area is likely heavily impacted.

Of particular interest is the relative timing of the increase/decrease in absorption, backscatter, chlorophyll-a and remote sensing reflectance relative to the commencement and subsequent cessation of dredging. Backscatter and remote sensing reflectance (at 412 nm) increase rapidly coincident with the start of dredging and then tend to show a slow return toward pre-dredging values to the end of the study period (December 2018). In contrast, the associated decrease in magnitude of both chlorophyll-a and absorption does not begin until after the cessation of dredging and neither shows a recovery to pre-dredging levels in the time period studied. It is thought that dredging activities initially stimulated phytoplankton growth providing nutrients to the surface ocean, but then decreased biological health of the region, evidenced with a decreased in absorption and chlorophyll concentration, by smothering natural benthic habitats and reef complexes with sediment.
